# Stiffer Spleen Predicts Higher Bone Marrow Fibrosis and Higher *JAK2* Allele Burden in Patients With Myeloproliferative Neoplasms

**DOI:** 10.3389/fonc.2021.777730

**Published:** 2021-10-26

**Authors:** Riccardo Moia, Micol Giulia Cittone, Paola Boggione, Giulia Francesca Manfredi, Chiara Favini, Bassel Awikeh, Anita Rebecca Pedrinelli, Abdurraouf Mokhtar Mahmoud, Maura Nicolosi, Mattia Bellan, Pier Paolo Sainaghi, Mario Pirisi, Gianluca Gaidano, Andrea Patriarca, Cristina Rigamonti

**Affiliations:** ^1^Division of Hematology, Department of Translational Medicine, Università del Piemonte Orientale and Azienda Ospedaliero-Universitaria Maggiore della Carità, Novara, Italy; ^2^Division of Internal Medicine, Department of Translational Medicine, Università del Piemonte Orientale and Azienda Ospedaliero-Universitaria Maggiore della Carità, Novara, Italy

**Keywords:** myeloproliferative neoplasms, spleen stiffness, *JAK2* mutations, vibration-controlled transient elastography (VCTE), NGS

## Abstract

A total of 63 myeloproliferative neoplasms [MPN; 9 polycythemia vera (PV), 32 essential thrombocythemia (ET), and 22 myelofibrosis (MF)] underwent spleen stiffness (SS) measurement by vibration-controlled transient elastography equipped with a novel spleen-dedicated module. Higher SS values significantly correlated with grade 2-3 bone marrow (BM) fibrosis (*p*=0.035), with hemoglobin level <10 g/dl (*p*=0.014) and with white blood cells ≥10,000/μl (*p*=0.008). Median SS was significantly higher in MF patients compared to ET and PV (*p*=0.015). SS also correlated with higher *JAK2* variant allele frequency (*p*=0.02). This study identifies SS as a potential noninvasive tool that reflects BM fibrosis and the mutational burden in MPN.

## Introduction

Spleen stiffness (SS) assessment by vibration-controlled transient elastography (VCTE) has emerged as a promising noninvasive tool with good diagnostic accuracy for predicting the degree of portal hypertension in patients with advanced chronic liver disease (1–5). Recently, SS and liver stiffness (LS) measurements have been investigated also in patients with BCR-ABL1 negative myeloproliferative neoplasms (MPNs), in which the spleen plays an active role (5, 6). Since SS has been shown to correlate with bone marrow (BM) fibrosis in patients with primary myelofibrosis (MF), it has been proposed that SS measured by VCTE might be used as a surrogate marker for BM fibrosis and as a predictor of clinical prognosis (5, 6).

However, these reports of SS assessment in MPNs have been conducted with a Fibroscan^®^ instrument that was not equipped with a spleen-dedicated module, which is now available and has higher accuracy than the liver-dedicated module (7, 8). Evidence was restricted to MF and did not include other BCR-ABL1 negative MPNs [i.e., polycythemia vera (PV) and essential thrombocythemia (ET)] that are also characterized by BM fibrosis, albeit at a lower grade (9, 10). Also, the correlation between SS and *JAK2* or *CALR* mutations was analyzed only with a qualitative assay that failed to detect a correlation (6).

In this study, we aimed at evaluating SS in patients affected by BCR-ABL1 negative MPNs by using the new Fibroscan^®^ 630 Expert instrument equipped with a spleen-dedicated module to assess in a more accurate manner whether SS has a potential role in predicting BM fibrosis, in correlation with quantitative mutational disease burden and disease severity.

## Methods

### Patients and Samples

Consecutive BCR-ABL1 negative MPN patients diagnosed according to the World Health Organization (WHO) criteria (9) followed at the Division of Haematology of Università del Piemonte Orientale between September 15 and October 15, 2020 were included in this cross-sectional study. All patients had BM biopsy performed at diagnosis and were provided with tumor genomic DNA (gDNA) isolated from peripheral blood (PB) and/or BM aspirates. SS was also evaluated in 45 healthy volunteers.

### VCTE Examination

Vibration-controlled transient elastography (VCTE) examinations for liver stiffness (LS) and spleen stiffness (SS) measurements were assessed using the instrument FibroScan^®^ 630 Expert (Echosens, Paris, France), equipped with liver (LSM@50Hz) and spleen dedicated (SSM@100Hz) modules coupled with an ultrasound localization system for the spleen. Results were expressed as kPa. LS and SS examinations were considered reliable only if at least 10 successful measurements were obtained, the success rate was at least 60%, and the interquartile range-to-median ratio (IQR/median) was ≤0.3. LS and SS measurements were performed by placing the patient in a supine position with the right and left arm, respectively, in maximum abduction and by placing the transducer in the right and left intercostal spaces, respectively. For SS measurement, the tip of the probe transducer was placed in a previously ultrasound targeted point in which the spleen parenchyma had been previously identified. LS and SS were assessed by two experienced operators. SS was also evaluated in 45 healthy volunteers using the FibroScan^®^ 630 Expert instrument.

### Mutational Analysis

gDNA isolated from PB or BM aspirates was analyzed for *JAK2*, *CALR*, and *MPL* by allele-specific PCR or by Sanger sequencing. gDNA was also subjected to next-generation sequencing (NGS) using the 54-gene TruSight Myeloid Sequencing Panel that provides allelic tumor burden of the mutations that are identified (**Supplementary Appendix**).

### Statistical Analysis

The Mann–Whitney test and the Pearson’s chi-squared test were used to compare clinical characteristics and gene mutations with spleen stiffness. Correlations between variables have been performed with Spearman’s rank correlation test. Statistical significance was defined as *p* value <0.05. Statistical analysis was performed using SPSS v.24.0.

## Results

A total of 65 BCR-ABL1 negative MPN patients underwent VCTE for the measurement of LS and SS. Measurement of SS was successful in 63/65 (97%) patients with a technique failure rate of only 3%. Median time for examination was 50 s (IQR 35–80). Among the 63 patients included in the study, the median age was 72 years (IQR 58–80), and 9 (14.3%) had a diagnosis of PV, 32 (50.8%) of ET, and 22 (34.9%) of MF. Among the 22 patients with MF, 5 patients had a previous history of ET and 6 a previous history of PV. The median hemoglobin level was 13.2 g/dl (IQR 11.8–14.3), the median white blood count was 6.5 x 10^3^/μl (IQR 4.8–9.2), and the median platelet count was 331 x 10^3^/μl (IQR 221–456). Splenomegaly, defined as a spleen longitudinal diameter higher than 12.5 cm, was present in 28 (44.4%) patients and, as expected, was more frequent in MF (72.7%) compared to PV and ET (29.3%) (*p*=0.001). Overall, 48 patients (76.2%) harbored *JAK2* mutations, 6 patients (9.5%) *CALR* mutations, and none had *MPL* mutations. Four (6.3%) patients were triple negative. The complete clinical and biological characteristics are reported in **Table 1**.

**Table 1:** Patients characteristics.

Characteristics	PV N=9	ET N=32	MF N=22	Total N=63
**Age (years)**	80 (64–85)	72 (58–81)	69 (59–76)	72 (58–80)
**Gender (No.)**				
Female	3 (33.3%)	16 (50.0%)	11 (50.0%)	30 (47.6%)
Male	6 (66.7%)	16 (50.0%)	11 (50.0%)	33 (52.4%)
**Arterial hypertension (No.)**	4 (44.4%)	16 (50.0%)	9 (40.9%)	29 (46.0%)
**BMI (kg/m^2^)**	28.4 (23.3–30.3)	25.2 (23.2–28.4)	24.0 (21.9–28.8)	24.8 (22.4–28.6)
**Disease duration (years)**	2 (1–8)	8 (3–13)	8 (4–18)	8 (3–13)
**Hb level (g/dl)**	13.3 (12.5–14.1)	13.5 (12.2–14.6)	12.7 (9.9–14.1)	13.2 (11.8–14.3)
**WBC (x10^3^/µl)**	6.5 (5.3–9.6)	6.1 (4.7–7.7)	7.1 (4.2–14.1)	6.5 (4.8–9.2)
**PLT count (x10^3^/µl)**	296 (226–374)	367 (262–535)	256 (122–378)	331 (221–456)
**Spleen LD >12.5 cm (No.)**	5 (55.6%)	7 (21.9%)	16 (72.7%)	28 (44.4%)
**Cirrhosis (No.)**	1 (11.1%)	0 (0%)	1 (4.5%)	2 (3.2%)
**Driver gene mutation (No.)**				
*JAK2*	9 (100%)	23 (71.8%)	16 (72.6%)	48 (76.2%)
*CALR*	0 (0%)	2 (6.3%)	4 (18.2%)	6 (9.5%)
*MPL*	0 (0%)	0 (0%)	0 (0%)	0 (0%)
Triple negative	0 (0%)	3 (9.4%)	1 (4.6%)	4 (6.3%)
**Liver stiffness (kPa)**	6.5 (5.8–7.2)	5.0 (4.0–6.0)	5.8 (4.0–6.3)	5.7 (4.5–7.2)
**Spleen stiffness (kPa)**	27.9 (22.0–33.3)	24.3 (21.1–28.4)	28.8 (25.6–36.3)	26.3 (22.3–33.6)
**Bone marrow fibrosis (No.)**				
Grade 0-1	8 (88.9%)	32 (100%)	10* (45.5%)	50 (79.4%)
Grade 2-3	0 (0%)	0 (0%)	12 (54.5%)	12 (19.0%)

PV, polycythemia vera; ET, essential thrombocytopenia; MF, primary myelofibrosis; No., number; BMI, body mass index; Hb, hemoglobin; WBC, white blood cells; PLT, platelets; LD, longitudinal diameter. Continuous variables are shown as medians with the interquartile range (IQR) in the round brackets. Categorical variables are shown as numbers (No.) with the % in the round brackets. *Include prefibrotic primary myelofibrosis.

The median SS was 26.3 kPa (IQR 22.3–33.6), and the median LS was 5.7 kPa (IQR 4.5–7.2). LS significantly correlated with SS (r=0.38, *p*=0.002) and body mass index (BMI) (r=0.40, *p*=0.001), but not with age, spleen longitudinal diameter, and BM fibrosis. SS correlated with BM fibrosis (r=0.36, *p*=0.005) and spleen longitudinal diameter (r=0.39, *p*=0.04), but not with BMI and age. Two PV patients with concomitant liver cirrhosis had the highest LS (39.0 and 14.5 kPa) and the highest SS (71.3 and 76.5 kPa) values in the cohort. In the 45 healthy volunteers, the median SS was 16.6 kPa (range 7.3–20.9 kPa).

**Figure 1:**
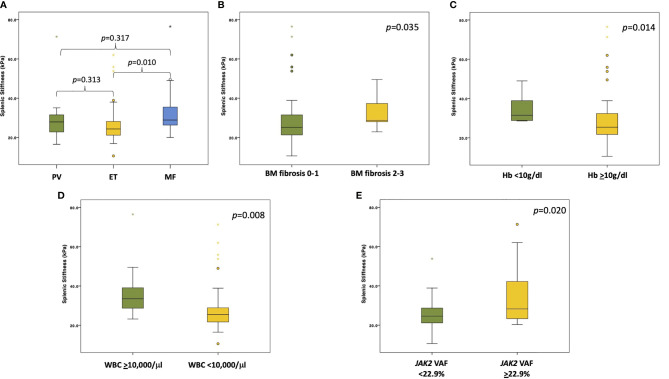
Boxplots showing different SS values stratified according to diagnosis and clinical/molecular variables. **(A)** The panel shows the SS values among the different MPNs (PV in green, ET in yellow, and MF in light blue). *p* values are reported above the boxplots. **(B)** The panel shows the difference in SS values between patients with grade 0-1 (in green) *vs* grade 2-3 (in yellow) BM fibrosis. *p* value is reported in the right upper corner of the panel. **(C)** The panel shows the difference in SS values between patients with Hb level <10 g/dl (in green) and patients with Hb level ≥10 g/dl (in yellow). *p* value is reported in the right upper corner of the panel. **(D)** The panel shows the difference in SS values between patients with WBC ≥10,000/μl (in green) and patients with WBC <10,000/μl (in yellow). *p* value is reported in the right upper corner of the panel. **(E)** The panel shows the difference in SS values between patients with *JAK2* VAF below the median value (in green) and patients with *JAK2* VAF above the median value (in yellow). *p* value is reported in the right upper corner of the panel.

By comparing SS across different MPN patients, SS was significantly higher in MF patients compared to ET and PV patients (*p*=0.015). More precisely, the median SS was significantly higher in MF (28.8 kPa; IQR 25.6–36.3) compared to ET (24.3 kPa; IQR 21.1–28.4) (*p*=0.01) (**Figure 1A**). No significant differences in the median SS were identified by comparing PV (27.9 kPa, IQR 22.0–33.3) *vs* ET (24.3 kPa, IQR 21.1–28.4) (*p*=0.313), and by comparing PV (27.9 kPa, IQR 22.0–33.3) *vs* MF (28.8 kPa, IQR 25.6–36.3) (*p*=0.317) (**Figure 1A**). Whereas the value of SS differed across MPN types, no differences in LS were observed among the three MPN subtypes analyzed. Even after excluding from the analysis the two PV patients with liver cirrhosis, all results for LS and SS assessment were superimposable across MPN subtypes.

The values of SS were then correlated with the major clinical variables of MPN. By stratifying all patients according to BM fibrosis, the median SS was significantly higher in those with grade 2-3 BM fibrosis compared to those with grade 0-1 fibrosis (28.7 kPa vs. 25.0 kPa, respectively; *p*=0.035) (**Figure 1B**). Conversely, LS did not significantly differ between patients with a higher or lower grade of BM fibrosis (*p*=0.842). In addition, SS significantly correlated with a lower hemoglobin level. In fact, the median SS value was 31.5 kPa (IQR 28.7–39.1) in patients with Hb <10 g/dl *vs* 25.4 kPa (IQR 21.7–32.8) in patients with Hb >10 g/dl (p=0.014) (**Figure 1C**). Also, SS significantly correlated with a higher WBC count, with a median SS of 33.5 kPa (IQR 27.5–41.7) for patients with WBC ≥10,000/μl *vs* 25.5 kPa (IQR 21.5–30.0) for patients with WBC < 10,000/μl (*p*=0.008) (**Figure 1D**).

A total of 51 patients underwent NGS analysis targeting 54 genes that are recurrently mutated in myeloid neoplasms. The median variant allele frequency (VAF) of *JAK2* was 22.9%. As expected, patients with a *JAK2* VAF above median (22.9%) had a significantly higher grade of BM fibrosis (*p*=0.003) compared to patients with a level of *JAK2* VAF below the median value. On these grounds, we proceeded to correlate the value of quantitative *JAK2* VAF with SS. Patients with *JAK2* VAF above the median value had a significantly higher SS (28.2 kPa; IQR 23.2–45.6) compared to patients with *JAK2* VAF below the median value (24.6 kPa; IQR 21.0–28.7) (*p*=0.027) (**Figure 1E**). Among the other nondriver genes analyzed by NGS, *TET2* mutated patients (3/51; 5.9%) showed a trend toward a higher SS compared to *TET2* wild-type patients (38.0 kPa vs. 25.2 kPa, respectively; *p*=0.118). At variance with NGS that provides a quantitative assessment of the mutational burden, a simple qualitative mutation assay by allele-specific PCR or by Sanger sequencing did not allow to identify correlations between *JAK2* (*p*=0.333) and *CALR* (*p*=0.692) mutations with SS.

## Discussion

In the present study, the employment of the novel FibroScan^®^ 630 Expert (Echosens, Paris, France), equipped with liver (LSM@50Hz) and spleen dedicated (SSM@100Hz) modules coupled with an ultrasound localization system for the spleen, allowed us to evaluate spleen stiffness in patients with MPN neoplasms in a user-friendly manner and to identify potential correlations with clinical and biological prognostic markers of MPNs.

Previous studies that aimed at evaluating SS in MPN patients were performed without a spleen-dedicated module leading to a successful rate of SS measurement of only 80% (5, 6). Because the spleen is physiologically stiffer than the liver, previous VCTE investigations with a liver-dedicated probe, rather than with a spleen-dedicated probe, led to overestimating the spleen stiffness (7, 8). To overcome these hurdles, a novel spleen-dedicated module based on VCTE has been recently developed (7, 8). In the present study, the successful rate of SS measurement was 97% suggesting that SS measurement with the spleen-dedicated module can overcome technical hurdles and represents a more accurate and reproducible tool to evaluate SS in MPN patients.

Previous reports describing the potential value of SS measurement in MPNs was mainly focused on MF (5, 6). In this study, which also included PV and ET, SS values did not overlap among the different MPN subtypes. Interestingly, MF patients harbored a high rate of SS compared to PV and ET suggesting that fibrotic stimuli in MF can involve different hematopoietic tissues including the spleen. We also subsequently evaluated the potential correlation between SS and different clinical and biological variables. Higher levels of SS correlated with a lower hemoglobin level, a lower platelet count, and higher WBC counts pointing to a more aggressive disease in patients with higher levels of SS. In our study, the median SS in 45 healthy volunteers was lower than the one measured in BCR-ABL1 negative MPN patients. These results are consistent with a previous report of SS measured in a group of 100 healthy volunteers with point shear wave elastography. In this group, the mean value of spleen stiffness was 18.14 (± 3.08) kPa, and statistical analyses showed no correlation between SS and sex, age, weight, and BMI (11).

MPN driver gene mutations represent an essential molecular marker in MPN diagnosis and prognosis, but their correlation with SS is not completely understood. By using Sanger sequencing or allele-specific PCR techniques, no correlations were found between the presence of *JAK2*, *CALR*, or *MPL* mutations with SS. This finding, assessed by qualitative molecular analysis, is consistent with what has been already reported in MF patients (6). However, allele-specific PCR and Sanger sequencing give dichotomous results and do not dissect the allele burden of gene mutations. Conversely, quantitative analysis defining the *JAK2* VAF identified a significant association of *JAK2* VAF with higher SS and, as expected, with higher levels of BM fibrosis. This finding reinforces the notion that *JAK2* allele burden correlates with a more aggressive disease characterized by a higher level of fibrosis not restricted to the BM but involving also the spleen (12).

In conclusion, since SS examination correlates with the degree of BM fibrosis and of *JAK2* allele burden, SS VCTE may provide a novel noninvasive and user-friendly assay complementing the analysis of histological and molecular biomarkers. Given the noninvasive nature of SS VCTE examination, this assay may be repeated multiple times in the individual patient, prompting longitudinal studies in larger cohorts aimed at defining the role of SS in prognostic models and in the long-term follow-up of MPN.

## Data Availability Statement

The original contributions presented in the study are included in the article/**Supplementary Material**. Further inquiries can be directed to the corresponding author.

## Ethics Statement

The studies involving human participants were reviewed and approved by CE 120/19. The patients/participants provided their written informed consent to participate in this study.

## Author Contributions

RM, MC, GG, AP, and CR designed the study, interpreted the data, and wrote the manuscript. PB, GM, CF, BA, ARP, AM, and MN performed VCTE examinations, performed molecular studies, and provided clinical data. MB, PS, and MP contributed to data interpretation and manuscript revision. All authors contributed to the article and approved the submitted version.

## Funding

This work was supported by Molecular bases of disease dissemination in lymphoid malignancies to optimize curative therapeutic strategies, (5 x 1000 No. 21198), Associazione Italiana per la Ricerca sul Cancro Foundation Milan, Italy; the AGING Project—Department of Excellence—DIMET, Università del Piemonte Orientale, Novara, Italy; and Ricerca Finalizzata 2018 (project RF-2018-12365790), MoH, Rome, Italy; Digital Microscopy project, Novara-AIL Onlus, Novara, Italy.

## Conflict of Interest

The authors declare that the research was conducted in the absence of any commercial or financial relationships that could be construed as a potential conflict of interest.

## Publisher’s Note

All claims expressed in this article are solely those of the authors and do not necessarily represent those of their affiliated organizations, or those of the publisher, the editors and the reviewers. Any product that may be evaluated in this article, or claim that may be made by its manufacturer, is not guaranteed or endorsed by the publisher.
