# Supplementary material for: Stiffer Spleen Predicts Higher Bone Marrow Fibrosis and Higher JAK2 Allele Burden in Patients With Myeloproliferative Neoplasms
Source: Front Oncol. 2021 Oct 26;11:777730. doi: 10.3389/fonc.2021.777730 (PMC8576346; doi:10.3389/fonc.2021.777730)
Supplement: Supplementary file 1 [file DataSheet_1.pdf]

SUPPLEMENTARY APPENDIX

Stiffer spleen predicts higher bone marrow fibrosis and higher *JAK2* allele burden in patients with myeloproliferative neoplasms

Supplementary methods

Allele-specific PCR and Sanger sequencing

The identification of *JAK2* (V617F) point mutation was achieved by allele-specific polymerase chain reaction (AS-PCR). AS-PCR was performed using two forward primers and one common reverse primer. The first forward primer for AS-PCR, which contains a mismatch on the third nucleotide of the codon 617 at the 3′-end, is specific to the mutant allele and produces an amplicon of 203 base pairs (bp). The second primer generates a 364bp product from both the mutant and wild-type alleles. Thirty-seven cycles of amplification with an annealing temperature of 58°C were performed. Amplicons were separated by size using 2% agarose gel electrophoresis in order to determine the presence of mutant alleles.

To detect *CALR* and *MPL* gene mutations, each patient's DNA was amplified by polymerase chain reaction (PCR) and sequenced by Sanger sequencing. The hotspot region of the two genes were amplified with specific primers: exon 9 for *CALR* and exon 10 for *MPL*. The amplified DNA was subjected to bi-directional Sanger sequencing. The sequence reaction was performed using the BigDye®Terminator v1.1 Cycle Sequencing kit (Applied Biosystem, Forest City, CA, USA). Sequencing analyses were performed on the ABI Prism 3130 Genetic Analyzer (Applied Biosystem, Forest City, CA, USA). The nucleotide sequences were analyzed both manually and by using the Mutation Surveyor software (version 4.0.8; Softgenetics, State College, PA, USA) which compares the sequence of the study sample with the germline reference sequence (reference Gene Bank NM_004343.3 for *CALR* and NM_005373.3 for *MPL*). All variants were coded according to the HGVS guidelines (www.hgvs.org/mutnomen/recs-DNA.html).

Next generation sequencing analysis

Tumor genomic DNA (gDNA) was analyzed using the 54-gene TruSight Myeloid Sequencing Panel (Illumina, San Diego, CA USA). At least 50ng of unfragmented gDNA were hybridized with a highly

multiplexed pool of oligonucleotide pairs to each region of interest (ROI). Each oligonucleotide contained a unique target-specific sequence and a universal adapter sequence, used in subsequent amplification reaction. For each sample, an extension-ligation reaction extended across the ROI and followed by ligation to unite the two probes to yield a library of templates with common ends. A polymerase chain reaction (PCR) amplified the library template and added specific indexes, sequence adapters to generate a single-stranded adapter-ligated libraries. After PCR clean-up, double-stranded DNA length and quantity of individual libraries were assessed by DNA 1000 kit and 2100 Bioanalyzer system (Agilent Technologies, St.Clara, CA, USA). Libraries were normalized according to the measured quantity and pooled in batches (30 libraries per pool). Paired-end sequencing runs were performed on a MiSeq with reagent kit v3 according to manufacturer's instructions. Sequence data generated from TruSight Myeloid libraries were analyzed using the DNA Amplicon app in Base Space™Sequence Hub. After FASTQ file generation the software utilizes a specially united Smith-Waterman aligner to adjust the peruses against the human hg 19 reference genome to generate BAM files and vcf files. The vcf files were analyzed using the Variant Interpreter app in Base Space™Sequence Hub. Among the variants, only protein truncating variants (i.e. indels, stop codons and splice site mutations), as well as missense variants not included in the dbSNP 138 and annotated as somatic in the COSMIC v92 database (https://cancer.sanger.ac.uk/cosmic), were retained.
